# A Sugar Gustatory Receptor Identified from the Foregut of Cotton Bollworm *Helicoverpa armigera*

**DOI:** 10.1007/s10886-012-0221-8

**Published:** 2012-12-06

**Authors:** Wei Xu, Hui-Jie Zhang, Alisha Anderson

**Affiliations:** CSIRO Ecosystem Sciences, Black Mountain, Australian Capital Territory 2601 Australia

**Keywords:** *Helicoverpa armigera*, Gustatory receptor, Foregut, Sugar receptor

## Abstract

*Helicoverpa armigera* (Hübner) is one of the most polyphagous and cosmopolitan pest species, the larvae of which feed on numerous important crops. The gustatory system is critical in guiding insect feeding behavior. Here, we identified a gustatory receptor from *H. armigera*, HaGR9, which shows high levels of identity to DmGR43a from *Drosophila melanogaster* and BmGR9 from *Bombyx mori*. Reverse transcriptase PCR (RT-PCR) revealed HaGR9 is highly expressed in larval foregut, with little or no expression in other chemosensory tissues. Membrane topology studies indicated that, like two previously studied *B. mori* GRs, BmGR8 and BmGR53, HaGR9 has an inverted topology relative to G protein-coupled receptors (GPCRs), an intracellular N-terminus and an extracellular C-terminus. Calcium imaging studies confirmed HaGR9 is a sugar receptor showing dose-dependent responses to D-galactose, D-maltose, and D-fructose. This highly-expressed foregut-specific gustatory receptor may contribute to the regulation of larval feeding behavior.

## Introduction

Insects display strong feeding preferences. The gustatory system plays a critical role in guiding insect feeding behavior. Gustatory stimuli from the environment are recognized by gustatory receptors (GRs), which are located in the gustatory sensilla distributed throughout the insect body. Despite a growing body of knowledge about the insect gustatory system, little is known about the molecular and cellular mechanisms that underlie recognition of gustatory signals.

Insect GR genes were first identified from *Drosophila melanogaster* genome based on a bioinformatics approach (Clyne et al., 2000). These proteins were found by using algorithms to search for seven-transmembrane domains, but they are strikingly different and share no sequence similarity with vertebrate GRs (Clyne et al., 2000). Furthermore, the topology is inverted compared to the classic G-Protein Coupled Receptors (GPCRs) (Benton et al., 2006; Robertson and Wanner, 2006; Zhang et al., 2011). Insect gustatory receptors have been classified into “GR43a-like” (Sato et al., 2011), “CO_2_” (Jones et al., 2007), “sugar” ( Dahanukar et al., 2001, 2007; Chyb et al., 2003; Jiao et al., 2007, 2008; Slone et al., 2007), and “bitter” clades (Wanner and Robertson, 2008; Lee et al., 2009). To date, much attention has been paid to the gustatory receptors of *Drosophila* (Dahanukar et al., 2001, 2007; Dunipace et al., 2001; Slone et al., 2007; Jiao et al., 2007, 2008; Gardiner et al., 2008; Lee et al., 2010, 2012;; Weiss et al., 2011), but with rapid progress of genome projects on other insect species such as *Anopheles gambiae* (Hill et al., 2002), *Bombyx mori* (Wanner and Robertson, 2008), *Tribolium castaneum* (Richards et al., 2008), *Apis mellifera* (Robertson and Wanner, 2006), and *Acyrthosiphon pisum* (Smadja et al., 2009), the research is extending to a diverse range of species. However, rarely are studies on gustatory receptors carried out on serious agricultural pests such as *Helicoverpa armigera*.

The cotton bollworm, *H. arimgera* (Hübner), is one of the most destructive insect species. The larvae feed on numerous important cultivated crops such as cotton, peanuts, soybeans, and maize. *Helicoverpa armigera* is distributed widely in Asia, Africa, Europe, and Australia, and causes approximately US$ 2 billion annual losses worldwide despite the use of insecticides (Sharm, 2001). The study of the *H. armigera* gustatory system may elucidate the underlying mechanisms that influence its feeding behavior and help to develop new insect-control strategies for such polyphagous pests.

In this study, we report the first gustatory receptor, HaGR9, from *H. armigera*. Further, we expressed HaGR9 in insect cells, and functionally characterized its topology and responses to substrates relevant to feeding behavior.

## Methods and Materials

### Insects and Cell Culture

*Helicoverpa armigera* were fed artificial food in the laboratory under conditions described previously (Akhurst et al., 2003). *Spodoptera frugiperda* Sf9 cells (Invitrogen, USA) were cultured in Sf-900 II SFM medium according to the manufacturer’s instructions. *Drosophila melanogaster* Schneider’s S2 cells were maintained as a suspension culture in *Drosophila* Schneider’s medium (Invitrogen, USA) adapting cells from 10 % fetal bovine serum (FBS, Invitrogen, USA) to 1 % FBS according to the Serum Halving Method. Cells were incubated at 28 °C and subcultured to a final density of 1 ~ 2 x 10^6^ cells/ml when they reached a density of ~6-10 x 10^6^ cells/ml (Mather and Roberts, 1998).

### RNA Isolation, cDNA Synthesis and PCR

Approximately fifty antennae, fifty mouthparts, ten foreguts, five midguts, and ten hindguts were dissected from mixed sex 5th instars. Approximately fifty tarsi and antennae were collected from male and female adults (d 1 – d 5). All collected tissues were stored immediately in RNA Later (Invitrogen, USA). Total RNA was purified using RNeasy (Qiagen, USA) or RNAqueous (Ambion, USA) kits according to the manufacturer’s protocol. The purified RNA was treated with DNase I, quantified, and qualified by a NanoDrop ND-2000 (Thermo Scientific, USA), and a 2100 Bioanalyzer (Agilent, USA). The cDNA was synthesized using a SMART RACE (Rapid Amplification of cDNA End) cDNA amplification kit (BD Sciences, Clontech, USA) with SuperScript II reverse transcriptase (Invitrogen, USA), according to the manufacturer’s manual. RT-PCR was performed for 40 cycles with an annealing temperature of 55 °C with primers (HaGR9-F CGCATGCTTTTATACTTAGG and HaGR9-R TCTCAATTGTCGTATCTTTGG). These primers spanned the known cDNA sequence and resulted in a 1300 bp fragment. 5' RACE PCR was performed according to the SMART RACE cDNA amplification kit manual with the universal primer and gene-specific primers (HaGR9-1 GCTGACCGTGAAGCCATTGCTGCGAG and HaGR9-2 CTTGAGATCCTAAGTATAAAAGCATGCGCTCCGC). PCR products were purified using QIAquick gel extraction reagents (Qiagen, USA), cloned into the pGEM-T Easy vector (Promega, USA) and sequenced.

### Immunocytochemistry

Sf9 or S2 cells were subcultured on poly-L-Lysine-coated coverslips in 6-well plates and transfected with 1 μg plasmid constructs and 6 μl of Fugene HD transfection reagent (Promega, USA) in 200 μl of medium per well. After 48 h post transfection, immunofluorescence under permeabilized and non-permeabilized conditions were performed as previously described (Zhang et al., 2011).

### Calcium Imaging

Sf9 cells were plated into 12-well plates and left to settle for 20 min before being transfected with 500 ng of plasmid construct (PIB/V5-His vector as control, HaGR9 or MYC-epitope tagged HaGR9) and 3 μl of Fugene HD transfection reagent (Promega, USA) in 100 μl of medium per well. After 48 h post transfection, calcium imaging and data analysis were performed using a modification of a previously described method (Zhang et al., 2011; Anderson et al., 2009).

### Tastants

The tastants tested were D-fructose, D-galactose, D-glucose, sucrose, D-maltose, D-trehalose, and *myo*-inositol with purities ≥ 99 %. D-galactose was purchased from Amresco (USA), whereas the others were purchased from Sigma-Aldrich (USA). The maximum final concentration of tastants in each well was 50 mM. For dose-dependent calcium imaging studies, D-fructose, D-galactose, and D-maltose were diluted with HBSS buffer (Zhang et al., 2011).

## Results

### Molecular Cloning and Expression Profile of HaGR9

**Fig. 1:**
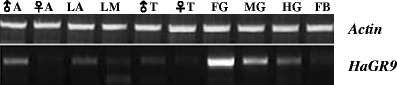
RT-PCR analysis of HaGR9 gene expression in adult and larval tissues of *Helicoverpa armigera*. ♂A, Male adult antennae; ♀A , Female adult antennae; LA, Larvae antennae, LM, Larvae mouthpart; ♂T, Male adult tarsi; ♀T, Female adult tarsi; FG, Foregut; MG, Midgut; HG, Hindgut; FB, Fat body. The *actin* gene was used as a control to qualify and quantify cDNA samples

Using DmGR43a amino acid sequence we searched in GenBank with Blastp (Altschul et al., 1990). A partial sequence of *H. armigera* olfactory receptor 4 (HaOR4) was found (EU818703) that contained 432 amino acids and lacked the N-terminal sequence. We studied its expression profile in several tissues: foregut, midgut, hindgut, antennae, and mouthparts from 5th-instars, as well as antennae and tarsi from male and female adults. PCR with gene-specific primers amplified a strong band of 1300 bp from the larvae foregut (Fig. 1). A weak band also appeared in male antennae, larvae antennae, midgut, as well as the hindgut. Interestingly, there was no expression seen in the female antennae. However when the number of PCR cycles was increased from 40 to 50 (data not shown), we were able to detect a weak band, thus suggesting there is a low level of expression in the female antennae. The cDNA sample from foregut was used in 5' RACE reactions to clone the full-length sequence.

**Fig. 2:**
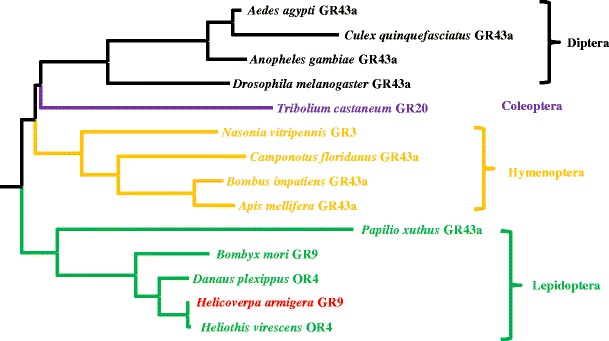
Phylogenetic analysis of GR43a-like receptor genes from insects. The analysed genes included: *Heliothis virescens* OR4 (CAD31946) (Krieger et al., 2002), *Danaus plexippus* OR4 (EHJ77681) (Zhan et al., 2011), *Bombyx mori* GR9 (NP_001124345) (Sato et al., 2011) (Wanner and Robertson, 2008), *Bombus impatiens* GR43a (XP_003486787), *Nasonia vitripennis*GR3 (NP_001164386) (Robertson et al., 2010), *Apis mellifera* GR43a (XP_001121326), *Drosophila melanogaster* GR43a (NP_523650) (Sato et al., 2011), *Tribolium castaneum* GR20 (EFA05758), *Anopheles gambiae* GR43a (XP_318100), *Camponotus floridanus* GR43a (EFN61344), *Aedes aegypti* GR43a (XP_001658898), *Culex quinquefasciatus* GR43a (XP_001842305), and *Papilio xuthus* GPCR (BAF91710) (Ozaki et al., 2011)

The full-length sequence was cloned and named HaGR9 (JX970522) because of its 69 % amino acid identity and 78 % similarity to BmGR9. The predicted protein contains 465 amino acids with seven predicted transmembrane domains (Split 4.0 Server, http://split.pmfst.hr/split/4/) (Juretic et al., 2002). It also showed 26 % amino acid identity and 49 % similarity to DmGR43a. We performed a phylogenetic analysis on HaGR9 with 13 other insect GR43a-like receptors (Fig. 2). These insect GR43a-like receptors were divided into 4 order-specific groups: Diptera, Coleoptera, Hymenoptera, and Lepidoptera (Fig. 2). To date, five GR43a-like receptors have been identified from Lepidoptera insects, *Papilio xuthus* (Ozaki et al., 2011), *Danaus plexippus* (Zhan et al., 2011), *B. mori* (Wanner and Robertson, 2008; Sato et al., 2011;), *Heliothis virescens* (Krieger et al., 2002) and *H. armigera*. Due to sequence homology exclusively with DmGR43a rather than any insect olfactory receptors, the two receptor genes from *H. virescens* and *D. plexippus* reported previously most likely have been misclassified as odorant receptor 4 (Krieger et al., 2002; Zhan et al., 2011). The phylogenetic analysis (Fig. 2) showed that GR43a-like genes are widely distributed in various insect species, including almost all species with a sequenced genome. We also found a GR43a-like protein from pea aphid, *A. pisum* (XP_003244306), but its sequence is much longer (756 amino acids) than all the other known GR43a-like receptors (~300-500 amino acids), and, therefore, we did not include it in our phylogenetic analysis (Fig. 2).

### Topology of HaGR9

**Fig. 3:**
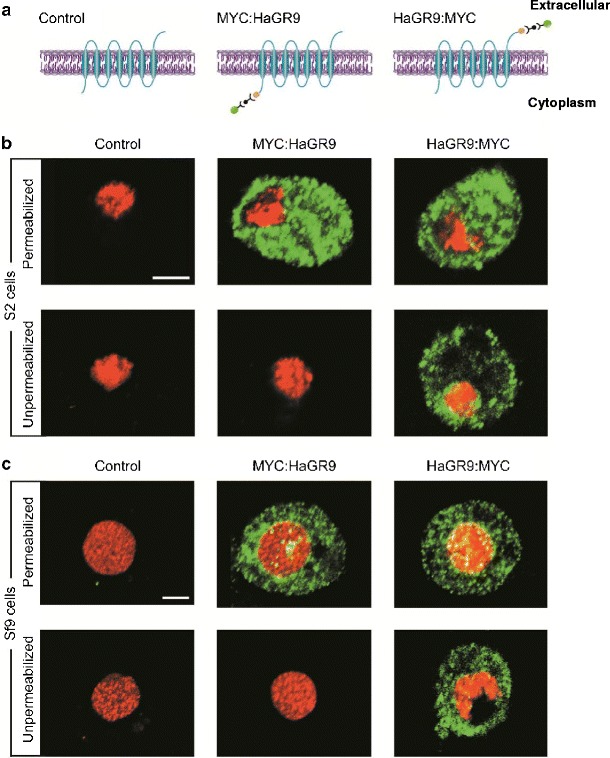
HaGR9 has extracellular C-terminus and intracellular N-terminus indicated by immunofluorescence in both Sf9 and S2 cells. HaGR9 was expressed in its native form or fused with two MYC-epitope tags at either the N- or C-terminus. **a** Expression constructs for native HaGR9 (controls), N-terminally MYC-epitope tagged HaGR9 (MYC:HaGR9), and C-terminally MYC-epitope tagged HaGR9 (HaGR9:MYC). **b**, **c** Immunofluorescence of HaGR9 in Sf9 and S2 cells under permeabilized and unpermeabilized conditions. Green indicates the location of MYC-epitope expression directed Alexa 488 fluorescence. Red indicates cell nuclear staining by DAPI. Scale bar = 5 μm

The algorithm Split4.0 predicts seven transmembrane domains for HaGR9 with an intracellular N-terminus and an extracellular C-terminus. To validate the topology we used immunofluorescence as previously described (Zhang et al., 2011). HaGR9 genes were fused to double MYC-epitope tags at either the N- or C-termini and expressed in both S2 cells and Sf9 cells. The native receptor (HaGR9) was used as a negative control. No positive signals were found from control cells (Figs.3b, c). Strong green fluorescence was visualized from cells transfected with either the N- or C-terminal labelled HaGR9 under permeabilized conditions (Figs. 3b, c). In contrast, green fluorescence could be detected only from cells transfected with C-terminally tagged HaGR9 (HaGR9:MYC) but not from cells transfected with N-terminally tagged HaGR9 (MYC:HaGR9) under unpermeabilized conditions. These results indicated that the N-terminus of HaGR9 is intracellular and the C-terminus is extracellular (Fig. 3a). The previous study of two BmGRs (BmGR8 and BmGR53 from the ‘sugar’ and ‘bitter’ clades) also showed that the N-terminus was inside the cell and the C-terminus located outside of the cell (Zhang et al., 2011), which is the same topology as observed for insect odorant receptors, but reverse to classical GPCRs (Benton et al., 2006). Insect receptor topologies previously were performed mainly on S2 cells from *Drosophila* (Smart et al., 2008; Zhang et al., 2011). Here, we used S2 cells (Fig. 3b) as well as Lepidoptera derived Sf9 cells (Fig. 3c). Both cell lines showed the same topology results, indicating both lines are suitable for insect receptor topology studies.

### HaGR9 Can Detect Three Sugars

**Fig. 4:**
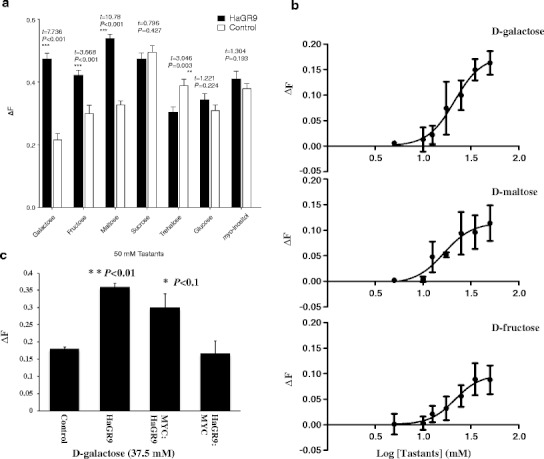
Responsiveness of HaGR9 to sugars. **a** Responses of HaGR9 to seven tastants at 50 mM. The mean responses of cells expressing HaGR9 are indicated by the dark solid bar. The mean responses of controls are indicated by the black hollow bars. Error bars indicate the standard error of the mean. Analysis of the statistical significance between each response and control was conducted by two-tailed Student’s *t*-test using arcsine transformation. *** *P* ≤ 0.001, **P < 0.01, *P < 0.05. **b** Log dose-dependent response curves for HaGR9 to D-fructose, D-galactose, and D-maltose. The average responses of control cells have been subtracted. Error bars indicate the calculated error of the difference between means = $SE\left( {{{\overline{x}}_{Gr12 }}-{{\overline{x}}_{control }}} \right)$. **c** Response of MYC-epitope tagged HaGR9 to 37.5 mM sugars. Control, empty vector (PIB/V5-His vector); HaGR9, HaGR9-transfected cells; MYC:GR9, two MYC-epitope copies fused to HaGR9 N-terminus; GR9:MYC, two MYC-epitope copies fused to HaGR9 C-terminus

We expressed HaGR9 in Sf9 cells and performed quantitative calcium imaging to characterize its specific ligand. Seven sugars, widespread in plant saps, and belonging to the monosaccharides (D-glucose, D-fructose, and D-galactose), disaccharides (sucrose, D-maltose, and D-trehalose), and *myo*-inositol were tested at 50 mM in the initial screen. Three of them, D-galactose, D-maltose, and D-fructose, generated responses that significantly differed from the control (Fig. 4a). A two-tailed Student’s *t*-test indicated that the order of responses of HaGR9 to three sugars is D-galactose (ΔF = 0.256, *t* = 7.74, *P* < 0.001) > D-maltose (ΔF = 0.211, *t* = 10.78, *P* < 0.001) > D-fructose (ΔF = 0.122, *t* = 3.67, *P* < 0.001). Our results also indicated that Sf9 cells themselves, although transfected with the empty expression vector, can be activated by the sugars, which we assume is due to endogenous receptors expressed in the Sf9 cell membrane. These responses vary based on the different sugars (Fig. 4a). Interestingly, cells transfected with HaGR9 showed reduced responses to trehalose when compared with responses from control Sf9 cells (Fig. 4a). We assume that the expression of HaGR9 may reduce or inhibit the expression, localization, or function of the endogenous taste receptors of Sf9 cells, thus leading to the lower responses to trehalose (Fig. 4a). Dose-dependent responses to D-galactose, D-maltose, and D-fructose were performed (Fig. 4b). Results showed that these three sugars can cause similar dose-dependent responses from 5 mM to 50 mM concentrations (Fig. 4b) with similar EC_50_ (21.3 ± 1.1 mM for D-galactose, 16.5 ± 1.3 mM for D-maltose, and 21.5 ± 1.1 mM for D-fructose).

In order to determine whether the modified GRs used for topology studies retained functionality, identical calcium imaging experiments were performed (Fig. 4c). Cells transfected with native HaGR9 and N-terminally labelled HaGR9 (MYC:HaGR9) both showed significant responses when compared to empty vector controls (Fig. 3b). However, there were no statistically significant differences in responses of C-terminally labelled HaGR9 (HaGR9:MYC) and the negative control. We cannot rule out the possibility that the labelled HaGR9 renders the protein non-functional due to the incorrect insertion of the receptor into the membranes. A previous study on BmGR8 showed similar results where the N-terminally labelled tag did not significantly affect the receptor’s function to detect *myo*-inositol (Zhang et al., 2011). However the C-terminally tagged receptor showed barely detectable function. These results indicate that the C-terminus of GRs is critical for their correct localisation and/or function.

## Discussion

Our phylogenetic analysis (Fig. 2) demonstrates that GR43a is a conserved GR that exists in almost all insect species (20-64 % amino acid identity), and therefore, GR43a may have a conserved function across insect orders. Our RT-PCR results (Fig. 1) indicate that HaGR9, a GR43a homologue, is highly transcribed in the larval foregut of *H. armigera*, where food is stored before moving into the midgut for digestion. A behavioral study on the blowfly, *Phormia regina*, showed the involvement of the foregut in the regulation of sugar taste threshold (Gelperin, 1966), with high sugar concentrations prolonging foregut stimulation and elevating the threshold for stimulation. Fructose and other sugars were shown to produce this behavioral effect when acting via the postulated foregut receptor (Gelperin, 1966). It is likely that HaGR9 may be a foregut receptor that is involved in the regulation of foregut stimulation and taste threshold in the digestive system, as well as in sugar sensing in external tissues. BmGR9, a GR43a ortholog from *B. mori*, also was shown to have high expression in the gut and respond to fructose significantly *in vitro* (Sato et al., 2011).

Recent studies in *Drosophila* show 14 gustatory receptors are expressed in the *Drosophila* gut, including GR43a, the fructose receptor, and GR64a from the sugar receptor family (Park and Kwon, 2011). The colocalization of the *GR-Gal4* drivers with the regulatory peptides neuropeptide F (NPF), locustatachykinin (LTK), and diuretic hormone 31 (DH31) also was observed in this study (Park and Kwon, 2011), providing further evidence that gustatory receptors may be involved in the detection and regulation of nutrients during digestion. Gastrointestinal chemosensation is an emerging field in humans and other mammals, where gustatory receptors expressed in the luminal epithelium are believed to be acting as nutrient sensors to guide digestive processes, to protect from harmful substances, and to regulate food uptake (Sclafani, 2007; Breer et al., 2012). Therefore, an improved understanding of the intestinal chemosensation mechanisms may assist in the development of novel treatments of eating disorders, obesity, diabetes, intoxication, and inflammation (Breer et al., 2012). Insect digestive systems have many similarities to those of vertebrates in basic structure, cell types, and development, and may provide a simple system to study intestinal chemosensation (Park and Kwon, 2011).

To study the topologies of HaGR9, we used both Sf9 and S2 cell systems. Both gave identical results, showing HaGR9, a member of the DmGR43a-like subfamily, has an intracellular N-terminus and an extracellular C-terminus. This result is consistent with previous studies on other insect gustatory receptors from the sugar and bitter subfamilies (Zhang et al., 2011) and also with odorant receptors (ORs) (Benton et al., 2006; Lundin et al., 2007; Smart et al., 2008), which have been shown to be an evolutionarily related family (Robertson et al., 2003). Previous studies suggest that the GR gene family is an ancient chemoreceptor family from which a branch of OR genes subsequently evolved (Robertson et al., 2003). Insect ORs are thought to function as odor-gated ion channels (Sato et al., 2008), although a modulatory role for G proteins and second messengers is likely to be involved in the downstream functions (Benton, 2008; Nakagawa and Vosshall, 2009). Here, we have shown that HaGR9 shares the same inverted topology as insect ORs, indicating that insect GRs are not classical GPCRs and may function in a way similar to insect ORs.

Genetic studies on *Drosophila* have revealed that coexpression of multiple GRs is essential for the detection of compounds like CO_2_, caffeine, theophylline, sucrose, D-glucose, and trehalose (Dahanukar et al., 2001,2007; Moon et al., 2006; Jones et al., 2007; Jiao et al., 2008 ). However, *in vitro* studies with BmGR8 (Zhang et al., 2011), BmGR9 and DmGR43a (Sato et al., 2011) have shown their responses to *myo*-inositol or D-fructose did not require the coexpression of other GRs, which is consistent with the HaGR9 studies reported here. We cannot conclude that HaGR9 can function without a co-receptor *in vivo* because Sf9 cells may express a native receptor that can assist HaGR9 in the detection of the three tested sugars. Orco, a highly-conserved OR co-receptor, was detected in Sf9 cells in a previous study (Smart et al., 2008), and is probably involved in the correct functioning of odorant receptors in the assay. In other *in vitro* studies using *Xenopus* oocytes, BmGR9 and DmGR43a showed a response only to D-fructose but not to other sugar tastants (Sato et al., 2011). HaGR9, the orthologous gene of BmGR9 and DmGR43a, showed responses to D-fructose, D-galactose, and D-maltose in our study. There may be two possibilities for these differences. First, HaGR9 shows 69 % and 26 % identity to BmGR9 and DmGR43a at the amino acid level, so it is possible the ligand binding capabilities differ. Second, Sf9 cells and *Xenopus* oocytes are different cell systems that may contain divergent components, leading to different activities and responses in the assay. To date, four major systems have been used to study insect chemosensory receptors: the “empty neuron” from *Drosophila* (Dobritsa et al., 2003), *Xenopus* oocytes with patch clamp technologies (Sakurai et al., 2004), human cells (HEK293T cells; Sato et al., 2008), and Lepidoptera cells (Sf9 cells) with calcium imaging (Smart et al., 2008; Anderson et al., 2009). We believe Sf9 cells are most suitable for studying Lepidoptera receptors as they are derived from a lepidopteran species, and therefore, are likely to more closely resemble *in vivo H. armigera* receptor function.

In summary, we here identified a novel insect-specific and insect-conserved gustatory receptor from *H. armigera,* and named it HaGR9. HaGR9 shows high homology to GR43a, and demonstrates high levels of transcription in the foregut of larvae. Topology study reveals this receptor has an intracellular N-terminus and an extracellular C-terminus, which is the same as previous studied insect GRs and ORs. In addition, HaGR9 shows dose-dependent responses to D-galactose, D-maltose, and D-fructose. Further immunohistochemistry studies to localize this receptor to specific cell types may shed light on the function of sugar receptors in the larval foregut. To study HaGR9 function *in vivo*, RNAi (Ozaki, et al., 2011) may be a powerful technique to inhibit its expression and perform feeding behavior assay. Studies on agricultural pests like *H. armigera* gustatory receptors may not only help us understand the underlying molecular mechanism of insect feeding behavior, but also help us develop new strategies to control this destructive species. For example, the studies on GR may direct the search for new attractants or repellents (Lee et al., 2010) from host plants that can be applied in pest control.
